# Encapsulation of Human-Bone-Marrow-Derived Mesenchymal Stem Cells in Small Alginate Beads Using One-Step Emulsification by Internal Gelation: In Vitro, and In Vivo Evaluation in Degenerate Intervertebral Disc Model

**DOI:** 10.3390/pharmaceutics14061179

**Published:** 2022-05-31

**Authors:** Sarit S. Sivan, Iris Bonstein, Yariv N. Marmor, Gadi Pelled, Zulma Gazit, Michal Amit

**Affiliations:** 1 Department of Biotechnology Engineering, Braude College of Engineering, P.O. Box 78, Karmiel 2161002, Israel; irisbon@braude.ac.il (I.B.); mamit@braude.ac.il (M.A.); 2 Department of Industrial Engineering and Management, Braude College of Engineering, P.O. Box 78, Karmiel 2161002, Israel; myariv@braude.ac.il; 3 Skeletal Biotech Laboratory, Faculty of Dental Medicine, The Hebrew University of Jerusalem, P.O. Box 12272, Jerusalem 91120, Israel; gadi.pelled@mail.huji.ac.il (G.P.); zulma.gazit@mail.huji.ac.il (Z.G.)

**Keywords:** microencapsulation, emulsification, internal gelation, alginate beads, mesenchymal stem cells, mammalian cells, intervertebral disc

## Abstract

Cell microencapsulation in gel beads contributes to many biomedical processes and pharmaceutical applications. Small beads (<300 µm) offer distinct advantages, mainly due to improved mass transfer and mechanical strength. Here, we describe, for the first time, the encapsulation of human-bone-marrow-derived mesenchymal stem cells (hBM-MSCs) in small-sized microspheres, using one-step emulsification by internal gelation. Small (127–257 µm) high-mannuronic-alginate microspheres were prepared at high agitation rates (800–1000 rpm), enabling control over the bead size and shape. The average viability of encapsulated hBM-MSCs after 2 weeks was 81 ± 4.3% for the higher agitation rates. hBM-MSC-loaded microspheres seeded within a glycosaminoglycan (GAG) analogue, which was previously proposed as a mechanically equivalent implant for degenerate discs, kept their viability, sphericity, and integrity for at least 6 weeks. A preliminary in vivo study of hBM-MSC-loaded microspheres implanted (via a GAG-analogue hydrogel) in a rat injured intervertebral disc model demonstrated long-lasting viability and biocompatibility for at least 8 weeks post-implantation. The proposed method offers an effective and reproducible way to maintain long-lasting viability in vitro and in vivo. This approach not only utilizes the benefits of a simple, mild, and scalable method, but also allows for the easy control of the bead size and shape by the agitation rate, which, overall, makes it a very attractive platform for regenerative-medicine applications.

## 1. Introduction

Cell encapsulation in microparticulate systems has been applied in a variety of biomedical processes and pharmaceutical applications, including the transplantation of pancreatic islet cells to reverse diabetes [1,2,3], the treatment of deficiency-related diseases and cancer [4,5,6,7], and also as microcarriers for drug delivery [8,9] and tissue engineering [10,11]. Cell encapsulation is generally used to protect cells from environmental stress while providing three-dimensional support [12,13,14,15]. Mesenchymal stem cells (MSCs) are recognized candidates for cell-based therapy and cell encapsulation owing to their biological traits [16,17]. They can be easily isolated from many types of tissues [18,19,20], expanded in vitro without maturation or differentiation, and genetically modified to express a variety of genes [21,22,23,24]. They are hypoimmunogenic and immunoregulatory and thus modulate immune responses [25,26], and their safety and feasibility have been proven in numerous clinical trials [27,28].

Alginate is a biocompatible biodegradable naturally occurring polysaccharide. It is a linear block copolymer that is composed of 1,4-linked-β-d-mannuronate (M-residues) and α-l-guluronate (G-residues), and alternating M and G residues that gel in the presence of divalent cations, such as calcium; in the case of G residues, a so-called “egg-box” structure is formed [29]. Commercial alginates are characterized by a broad range of M/G ratios and viscosity levels. Alginates extracted from different sources (brown alga or bacterial) have variable properties, and beads that are produced using different crosslinking methods possess a wide range of physical and biological properties that affect the cell response in vitro and in vivo [30]. Due to the resemblance to the natural extracellular matrix and the relatively mild crosslinking conditions of preparation, alginate hydrogels have been employed as scaffolds for tissue engineering [31,32], and also for the encapsulation of transplanted cells [32,33], as they provide an immunoisolating barrier for cells.

Alginate beads can be formulated through a wide range of techniques, including extrusion, coaxial air or liquid flow, vibrating jet cutters, and electrostatic potential [9], by using an external source of calcium. Extrusion is a very popular technique. It utilizes an external source of alginate and can be applied using simple dripping (through a syringe), or by using electrical, mechanical, or jet-cutting mechanisms to control the particle size. The electric field generates beads with diameters in the range of 500–2000 µm [34]. While beads formed using mechanical vibration have diameters in the range of from 300 µm to 5 mm [35], the lower bound of those produced with jet cutting is shifted to a 200 µm diameter [36]. Despite the control over the size, these methods are often complex and time consuming [9,37]. Although the simplest method for producing uniform alginate gel particles is extrusion by a syringe, the resulting particles are large and require time to cure in the gelling bath. This method is also limited to alginate solutions with low viscosity (<200 cP), due to difficulties in pumping and needle blockage. Furthermore, these methods are only suitable for small volumes of biopolymer, and, despite the attempts made, these methods have not realized industrial scale-up [37]. Other techniques utilize microfabricated channel arrays [38,39] or microfluidic systems [40,41] for the formation of small (50–200 µm) monodispersed size-controlled beads; despite the monodispersity and the control over the bead size and shape, the scalability of these methods is limited.

Internal gelation, which was first developed by Poncelet et al. [42] to immobilize DNA, proteins, and bacteria [43,44,45], and was later adapted for mammalian-cell encapsulation [46,47], involves the dispersion of an insoluble (or slowly soluble) calcium complex in the Na-alginate solution. Upon pH reduction below the pK_a_ of the uronic acid residues, Ca^2+^ ions are released from the calcium complex, crosslinking the alginate to form a homogeneous hydrogel [48], which is stabilized by intermolecular hydrogen bonding [49]. This method facilitates encapsulation in a stirred vessel by using a single-step protocol instead of the drop-by-drop approach produced by conventional bead generators. Control over the gelation process has enabled the design of particle morphologies and matrix density, allowing the production of a wide range of bead sizes under various process conditions [50]. Moreover, the liberation of carbon dioxide facilitates the formation of a more porous and looser gel matrix compared to those obtained via external gelation. These features are advantageous in the encapsulation of cargo that requires an efficient solute exchange with the external environment, such as living cells and enzymes for tissue engineering or biocatalytic applications. Scaling up has been shown to be possible [42], and the method is suitable for small-to-large-scale production over a broad range of alginate concentrations. It is inexpensive, readily available, and rapid.

Generally, a bead size in the range of 200–1000 µm is used for biomedical applications. However, encapsulation methods require further optimization to reduce the size while maintaining stable structures. Small beads (<300 µm) offer many advantages for transplanted cells, including more rapid mass transfer (due to a large surface-to-area ratio) [51], less intense fibrosis reaction [52], better mechanical strength, and easier implantation, compared to larger particles [53]. Small beads can also be administered by using minimally invasive techniques [54].

Various groups have produced small microspheres using different methods. Landazuri et al. [55] and Khatab et al. [56] encapsulate MSCs using an electrostatic encapsulator equipped with a nozzle. Despite the high viability obtained, the main drawback of these methods is their laboratory-scale apparatus. Small and monodispersed microspheres were also produced using internal gelation adapted for a microfluidic reactor [57,58,59]. In these studies, either poor cell encapsulation (not all the beads contained cells) or small deformed beads containing a high percentage of fragmented cells can be seen. However, the main drawback is the limited throughput of microparticles fabricated by microfluidics [60]. Hoesli et al. [46,47,48] have tried but were unable to produce small alginate cell-encapsulating microspheres by internal gelation using conventional emulsification methods.

The primary objective of this study is to produce small (<300 µm) alginate beads that are capable of encapsulating mammalian cells and maintaining their long-lasting viability by using one-step emulsification by internal gelation. This study presents data for a range of hBM-MSC-encapsulating alginate beads that vary in size, as controlled by the agitation rate. Three formulations were prepared and characterized for their viability in vitro and in vivo using a rat-caudal-intervertebral-disc model.

## 2. Materials and Methods

### 2.1. Materials

l-glutamine, penicillin/streptomycin solution, trypsin-EDTA solution B, and sodium pyruvate solution were purchased from Biological Industries (BI), Beit HaEmek, Israel. Fetal bovine serum (FBS) was from Gibco, Life Technology, Grand Island, NY, USA. Alginate (A2033, medium MW 3.5 × 10^5^ g/mol, M/G ratio: 1.56 [61]), propidium iodide (PI), fluorescein diacetate (FDA), HEPES Sodium 2-acrylamido 2-methyl propane sulfonic acid (NaAMPS) (58% in aqueous solution), polyethylene glycol diacrylate (PEG575DA, Mn ~500), ascorbic acid (AA), potassium peroxymonosulfate (Oxone), polyethylene glycol diacrylate (PEGDA), and 3-Sulfopropyl acrylate potassium salt (KSPA) were purchased from Sigma, Rehovot, Israel. Acetic acid glacial was from Carlo ERBA, Val-de-Reuil, France. Calcium carbonate (CaCO_3_) was purchased from DAEJUNG, Siheung-si, Gyeonggi-Do, Korea. Dil (1,1′-dioctadecyl-3,3,3′3′-tetramethylindocarbocyanine perchlorate) Cell-labeling solution and Live/Dead™ Cell Imaging kit were purchased from Invitrogen™, Waltham, MA, USA. Live Cell Imaging Solution was from Molecular Probes™, Eugene, OR, USA.

### 2.2. Cell Line

Human-bone-marrow-derived mesenchymal stem cells (hBM-MSCs) (ATCC, Manassas, VA, USA) were cultured in a dedicated complete growth medium: MEM-alpha (Biological Industries, Beit HaEmek, Israel), supplemented with 10% (*v*/*v*) FBS and 1% (*v*/*v*) penicillin/streptomycin.

### 2.3. Preparation of Cell-Loaded Alginate Beads

Cells were immobilized in alginate beads in a stirred flat-bottomed vessel by a simple one-step method, as previously reported [48]. Beads containing medium-viscosity high-mannuronic (MVM) alginate (1.7%, *w*/*v*) were prepared, given that high cell viability has been reported in the range between 1.2% and 2% (*w*/*v*) alginate [40,41,62]. Briefly, a complete growth medium (1.1 mL) containing 10.5-fold concentrated cells (2.3 × 10^7^ cells) was mixed with alginate solution (9.9 mL, 2% *w*/*v*) in HEPES buffer (10 mM HEPES and 170 mM NaCl, pH 7.4) and CaCO_3_ (550 µL, 24 mM in HEPES buffer). The alginate mixture was then added to mineral oil (10 mL) to create an emulsion, and agitated using one of three rates (800, 900, or 1000 rpm) for 12 min. Internal gelation was achieved by an acidification step using mineral oil (10 mL) with acetic acid (0.4%, *v*/*v*), and the mixture was allowed to agitate for an additional 8 min. Beads were recovered by phase inversion, first by adding HEPES buffer (40 mL) with complete medium (10%, *v*/*v*), and agitated at a low rate (~500 rpm) for 1 min, followed by centrifugation (630× *g* for 5 min). Beads were collected following aspiration of the oil phase and washed with the complete medium through a 70 µm mesh strainer. Recovered microemulsions were ready to be cultured.

### 2.4. Yield and Encapsulation Efficiency

The yield of the overall encapsulation process was determined as:

$$\mathrm{Yield}\;(\mathrm{volume}\;\%)=\;\frac{\mathrm{Total}\;\mathrm{volume}\;\mathrm{of}\;\mathrm{beads}\;\mathrm{formed}\;}{\mathrm{Starting}\;\mathrm{volume}\;\mathrm{of}\;\mathrm{alginate}}\;\cdot 100$$

The encapsulation efficiency (EE) was calculated as follows:

$$\mathrm{EE}\;(\%)=\;\frac{\mathrm{number}\;\mathrm{of}\;\mathrm{encapsulated}\;\mathrm{cells}\;}{\mathrm{total}\;\mathrm{number}\;\mathrm{of}\;\mathrm{cells}\;\mathrm{initially}\;\mathrm{used}}\;\cdot 100$$

The total volume of the beads formed was assessed by measuring the excluded volume after transferring the beads to a known volume. The number of encapsulated cells was assessed directly by the Trypan blue exclusion method, following recovery of the cells by degelling [48].

### 2.5. Viability Measurement

Cell-loaded beads were cultured in a complete medium at 37 °C with 5% CO_2_. For beads prepared at different agitation rates, cell viability was assessed at predetermined time intervals (1, 7, and 14 days), using the dual-staining assay. Briefly, cells were stained using dual-staining solution containing 16 µM FDA and 30 µM PI in Live Cell Imaging Solution. A sample of cell-containing beads was mixed with HEPES buffer, which contained 10% (*v*/*v*) complete medium. The staining solution was then added to the sample (1:1, *v*/*v*) and incubated for 40 min on ice. Images were captured by the Apotome 2 microscope (Zeiss, Jena, Germany), using Filter Set 38 HE (Ex/Em: 470/525) for FDA and Filter Set 63 HE (Ex/Em: 572/629) for PI. For beads embedded within GAG analogues, cell viability was assayed by the Live/Dead assay kit.

### 2.6. Recovery of Cells from Beads

Cells were recovered from the beads, after a wash step through a 70 μm mesh strainer, using a degelling solution (55 mM sodium citrate, 30 mM EDTA, and 150 mM NaCl at pH 6.8) diluted with 9 volumes of MEM medium. Six volumes of the diluted de-gelling solution were further diluted with one volume of beads for 2 min at room temperature to allow recovery of cells from the beads before quantification with the Trypan blue exclusion method.

### 2.7. Morphology and Size Distributions

Bright-field images were used to determine bead morphology and distribution of bead diameters, using the NIS-Elements module (Nikon, Brighton, MI, USA).

### 2.8. Preparation of Glycosaminoglycan (GAG)-Analogue Hydrogel

GAG analogues were prepared using sulphonate-containing monomers polymerized in the presence of a crosslinking agent, utilizing redox polymerization. Hydrogels were formed using a typical two-part pre-gel component, in which Part (a) contained water, oxone (redox initiator), and KSPA monomer, and Part (b) contained water, AA (redox initiator), NaAMPS monomer, and PEGDA (crosslinker), as previously detailed [63]. A crosslinking density (concentration of PEGDA) of 1.5% was used. To form a hydrogel, equal volumes of components (a) and (b) were placed on separate sides of a double-barreled syringe system (Plas-Pak Industries, Inc., Norwich, CT, USA), simultaneously injected into the required cavity, and allowed to settle.

### 2.9. In Vivo Studies

The animal study protocol was approved by the Ethics Committee of the Hebrew University (MD-17-15342-4). Cell-loaded alginate beads were implanted in a damaged disc model using a biomimetic glycosaminoglycan (GAG) hydrogel [63] as a carrier. The viability of implanted cells was assessed 8 weeks post-procedure.

#### 2.9.1. Preparation of Cell-Loaded Alginate Beads for Transplantation

Cell-loaded alginate beads were prepared as previously described in Section 2.3. For the in vivo studies, beads prepared at 900 rpm were used. Prior to encapsulation, cells were predyed with CM-Dil. Briefly, 10^6^ cells/1 mL serum-free medium were incubated with 5 μL/mL CM-DiI at 37 °C for 5 min, followed by 15 min incubation at 4 °C, and finally washed with PBS. Cells were encapsulated in alginate beads and cultured in a differentiating medium [64]. Cell-loaded and empty (as control) alginate beads (10^6^/mL beads) were further immobilized in a GAG-analogue hydrogel [63]. Briefly, 10% (*v*/*v*) of cell-loaded beads were mixed with hydrogel solutions (containing 1.5% PEGDA) and allowed to settle in vitro using a silicone tube with an inner diameter of 0.7 mm. The cured ‘spaghetti’-like gel was cut to cylinders of 1 mm height, which were allowed to swell for 4 days in PBS. The swollen cylinders (1.5 mm in diameter and 1.5 mm in height) were used for transplantation.

#### 2.9.2. Rat Surgical Procedure

Nine female Wistar rats (~200 g) were used, of which six animals were operated on. Before surgery, each animal was anesthetized by inhaling isoflurane; the use of anesthetic gas allows for faster recovery of the animals. After having the animal anesthetized, tails were cleaned with a surgical scrub, and a longitudinal incision of up to 3 cm long was made to expose the three most cranial tail discs. In each disc, using a blade, the nucleus pulposus was removed using a curette, and one GAG-analogue-hydrogel cylinder containing cell-loaded alginate beads was implanted into the nucleus pulposus cavity. The annulus was then closed with two 5.0 nonresorbable nylon sutures, and, finally, the tail skin was closed using resorbable sutures. Following the procedure, rats were allowed unrestricted activity in separate cages and were monitored for signs of pain or infection. Buprenorphine (0.01–0.02 mg/kg) was administered as needed for pain. At 8 weeks post-surgery, animals were euthanized. Tails were harvested and fixed in 4% formalin and processed for histology using hematoxylin and eosin (H&E) and 4′,6-diamidino-2-phenylindol (DAPI).

## 3. Results and Discussion

### 3.1. Choice of Alginate for Bead Production

High-M alginate was chosen (M/G of 1.56), as it exhibited lower permeability compared to high-G alginate [65]. It is also of particular practical interest for cell transplantation because high-M alginates are usually less viscous, allowing gels with a higher alginate content to be made. The cell-encapsulating beads were prepared at different agitation rates (800–1000 rpm) and were expected to yield bead diameters between 100 and 300 microns [48]. As can be seen, the encapsulation efficiency of the process was not affected by the agitation rate (Table 1).

### 3.2. The Effect of Agitation Rate on Bead Diameter

The effect of the agitation rate on the bead diameter is summarized in Table 2. The data clearly show that the bead size can be controlled by the agitation rate.

**Figure 1:**
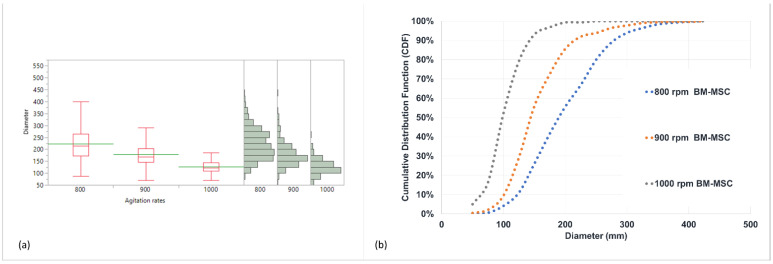
Characterization of alginate beads: (**a**) size distributions of hBM-MSC-loaded alginate beads formed at agitation rates of 800, 900, or 1000 rpm; and (**b**) corresponding cumulative-distribution-function (CDF) plot of bead diameter at different agitation rates.

The average diameter and respective size distribution (represented by the standard deviation) decreased significantly with an increase in the agitation rate (*p* < 0.0001), as is also shown in Figure 1a. As is clearly demonstrated by the cumulative distribution function in Figure 1b, in the case of lower agitation rates, not only the average size is bigger, but also all of the respective percentiles. This is most likely due to the higher shear that is imposed on the emulsion and, subsequently, on the beads formed, either empty (data not shown) or cell-loaded.

### 3.3. In Vitro Viability of Cells Encapsulated in Alginate Beads

The encapsulation of cells using alginate has been extensively investigated; generally, the main challenge is to maintain the long-lasting viability of encapsulated cells in vitro and in vivo.

**Figure 2:**
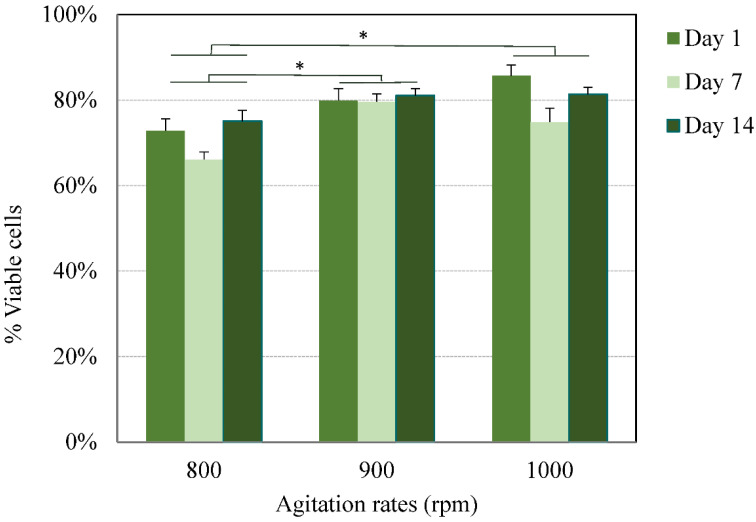
Viability of hBM-MSCs encapsulated in alginate beads prepared at various agitation rates. Cells were encapsulated in MVM alginate beads (2 × 10^6^ cells/mL alginate) at agitation rates of 800, 900, and 1000 rpm and cultured for up to two weeks. Cells were recovered from the beads following degelling and counted using the Trypan blue exclusion method. For any agitation rate, no significant difference was noted in the viability between the different days (*p* < 0.05, linear regression model). No statistical difference was noted in the average viability between 900 and 1000 rpm (* *p* < 0.01, Tukey test).

**Figure 3:**
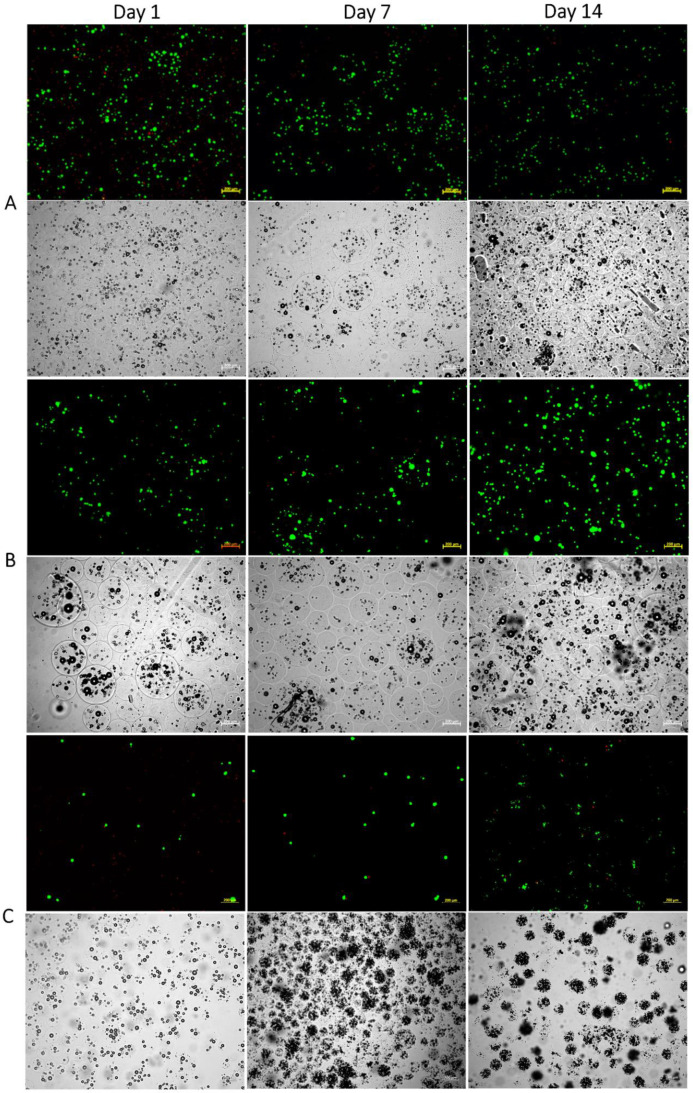
Microscope photographs of alginate beads containing hBM-MSCs observed in fluorescence and bright-field modes. hBM-MSCs were encapsulated in MVM alginate beads (2 × 10^6^ cells/mL alginate) at agitation rates of (**A**) 800, (**B**) 900, and (**C**) 1000 rpm, and cultured for up to 2 weeks. For the fluorescence mode, cells were stained with FDA/PI. The scale bar is 200 µm.

**Figure 4:**
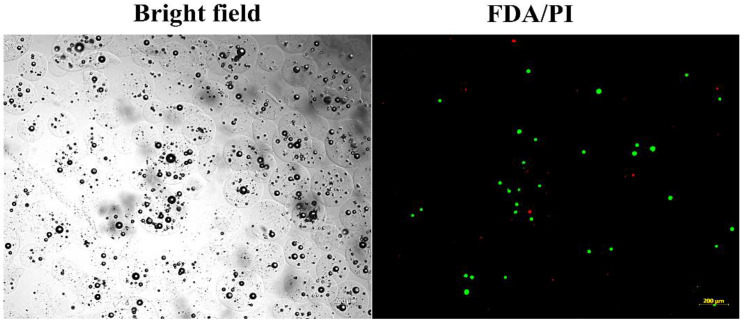
Microscope photographs of alginate beads containing hBM-MSCs observed in fluorescence and bright-field modes. hBM-MSCs were encapsulated in MVM alginate beads (2 × 10^6^ cells/mL alginate) at agitation rates of 900 rpm and cultured for up to 4 weeks. For the fluorescence mode, cells were stained with FDA/PI. The scale bar is 200 µm.

In the current study, following the exposure of cells to the acidic conditions that resulted from the method used, all three formulations were first monitored for their viability for up to 2 weeks. The viability of the encapsulated cells at various agitation rates was assessed by the Trypan blue exclusion method at predetermined time points (1, 7, and 14 days) following bead degelling. The average viability for all agitation rates, throughout the period tested, was estimated at 75 ± 7% and 81 ± 4% for beads obtained at the higher agitation rates (Figure 2). Subsequently, the viability of cells encapsulated in beads prepared at 900 rpm, which are most suitable in terms of their size, was further tested 4 weeks post-encapsulation. The cells maintained their viability for the period tested. Fewer small clusters were noted at 900 rpm, which did not have any significant effect on the beads’ integrity or viability (Figure 3 and Figure 4).

Despite the polydispersity obtained in the bead size, which is typical of emulsion techniques, the average viability at each agitation rate throughout the period tested was reproducible. The total number of cells (live and dead) counted throughout the period tested remained unchanged, suggesting that no proliferation occurred. However, further studies are required to test the long-term effect on the fate of encapsulated hBM-MSCs.

As seen in Figure 4, the beads obtained were round and remained intact for at least 4 weeks, as shown by the bright-field images. The bead sphericity could be explained by the fact that gelation is initiated from within the droplet, and the insoluble calcium salt is dispersed evenly within the alginate droplet, thus forming a more uniform polymer distribution across the dispersed droplet upon acid introduction [66], unlike external gelation, where the addition of calcium may deform the shape of the beads. The stability of high-M structures may be promoted by both ionic gelation, whereby the calcium crosslinks prioritize the guluronic block, and by acidic gelation, whereby, due to local regions of low pH [37], mannuronic acid participates in hydrogen-bond formation.

Previous studies using methods based on external gelation produced small beads with a short-term high-viability rate in vivo and in vitro [55,56,62]. When considering the viability rate using methods based on internal gelation adapted to microfluidic devices, Workman et al. [59] report a sharp decrease in the viability of human embryonic kidney cells (HEK239) 3 weeks post-encapsulation in vitro, after which the viability increased to ~70% for up to 90 days, for all the populations tested. Tan et al. [58] report the production of small monodispersed alginate beads with a viability of up to 74.3% immediately following encapsulation. No data are available for the viability of cells in small alginate beads using conventional emulsification by internal gelation. Hoesli et al. [46,47,48] have tried but were unable to produce small alginate cell-encapsulating microspheres using this method. This can be attributed to the composition of the alginate used (M/G of 1 compared to 1.56 used in this study). In these studies, large beads (757 ± 20 µm) with a cell viability of 71 ± 4% for primary cells immediately after encapsulation were obtained. However, 10 days later, the viability decreased to 35 ± 6% of the initial number of cells seeded.

### 3.4. In Vitro Viability of Bead-Encapsulated Cells Seeded within GAG-Analogue Hydrogel

**Figure 5:**
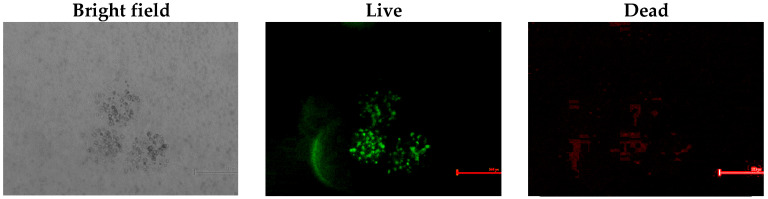
Viability of hBM-MSCs encapsulated in alginate beads after seeded in GAG-analogue hydrogel. Constructs comprising GAG analogue (1.5%) and encapsulated cells (2 × 10^6^ cells/mL beads) were incubated for six weeks and stained with a LIVE/DEAD^®^ assay kit. hBM-MSC-loaded beads were imaged using bright-field and fluorescence microscopy. The scale bar is 200 µm.

The ability of beads to protect cells from an acidic environment and maintain their viability was tested by using GAG-analogue hydrogels, which were previously proposed as mechanically equivalent implants for degenerate intervertebral discs [63]. Due to the sulphonate-containing moieties, these GAG analogues possess an acidic environment. In our previous tests, bare hBM-MSCs seeded within these GAG-analogue hydrogels did not survive (data not shown). To overcome this, the cells were first encapsulated in alginate beads and were subsequently seeded within GAG-analogue hydrogels, after which they were stained for their viability. The cells maintained their viability for at least six weeks in the GAG-analogue hydrogel, as seen by the fluorescent live/dead staining (Figure 5), and also their sphericity, as can be seen by the bright-field images.

### 3.5. In Vivo Viability of Cell-Loaded Beads Transplanted into an Intervertebral Disc Model

**Figure 6:**
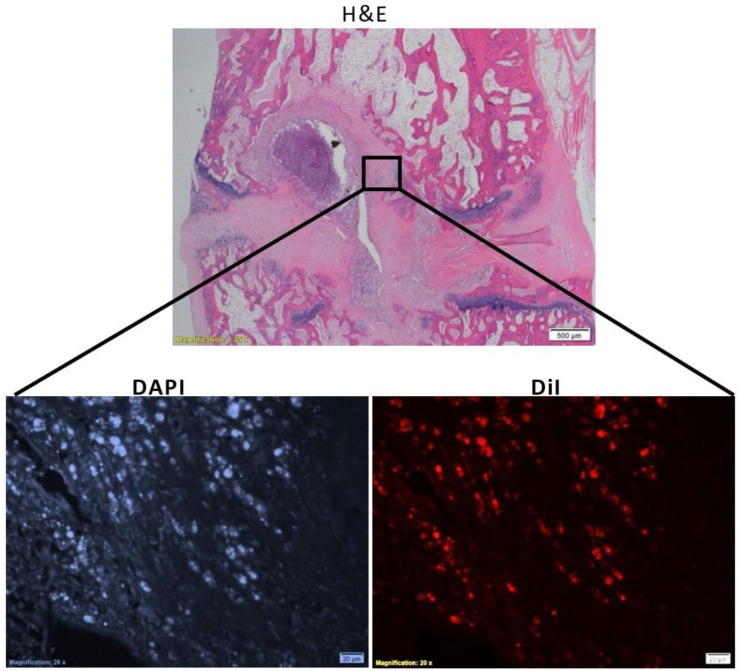
Cross section of a rat intervertebral caudal tail disc with damaged nucleus pulposus 8 weeks post-implantation of hBM-MSC-loaded alginate beads embedded in GAG-analogue hydrogel. Tails were harvested, fixed in 4% formalin, and stained with H&E and DAPI. Cells were stained with Dil (live staining) before seeding in alginate beads. The scale bar is 20 µm.

**Figure 7:**
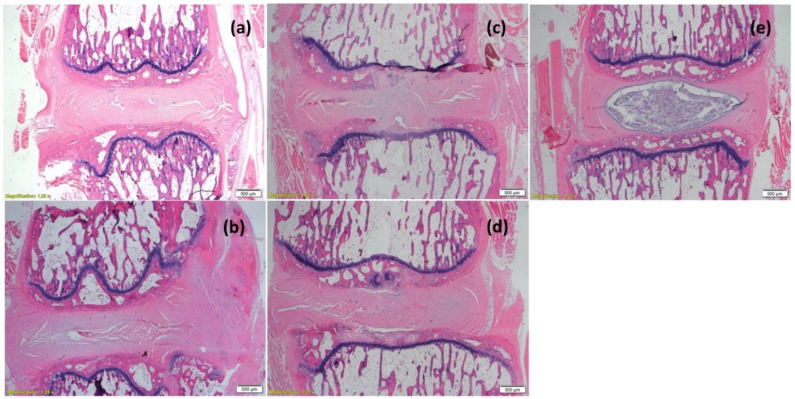
Cross section of two adjacent rat intervertebral caudal tail discs with damaged nucleus pulposus, 8 weeks post-implantation with 1.5% GAG-analogue hydrogel, showing: (**a**) empty hydrogel (Rat #1); (**b**) hydrogel with empty alginate beads (Rat #3); (**c**) hydrogel with hBM-MSCs encapsulated in alginate beads (Rat #5). Moreover, (**d**) a disc following nucleotomy (Rat #5), and (**e**) intact disc (Rat #7), are presented. Tails were harvested, fixed in 4% formalin, and stained with H&E. Scale bar is 500 µm.

The capability of the construct (cell-loaded beads seeded in GAG analogue) to maintain cell viability in vivo was further tested. To this aim, constructs were implanted in an intervertebral-caudal-disc model with a damaged nucleus pulposus as a preliminary proof of concept for their long-term biocompatibility and viability in vivo. As is seen in Figure 6, the implanted cells survived 8 weeks post-transplantation within the discs, as detected by DiI fluorescence, on the background of resident cells, as stained by DAPI. No detectable beads were seen 8 weeks post-implantation. Hydrogels with and without beads (empty and cell-loaded) were well tolerated, and no immune reaction was detected (Figure 7). The competence of the proposed system as an attractive platform for regenerative-medicine applications is further supported by the fact that the cell survived 8 weeks through two physical barriers (the beads’ walls and the hydrogel).

## 4. Conclusions

This study sought to utilize the benefits of emulsification by internal gelation to form small alginate beads that are capable of maintaining the long-lasting viability of hBM-MSCs. Here, we demonstrate that small-sized (<300 µm) medium-viscosity high-M alginate beads, formed by one-step emulsification using internal gelation, can be used as a microencapsulating system for hBM-MSCs in vitro and in vivo. The present method has been shown to have minimal effect on the viability of encapsulated cells during and post-processing, both in vitro and in vivo. The encapsulated cells maintained their viability in vivo in a rat caudal model, and, thus, they can be potentially used for the treatment of degenerate intervertebral discs, as well as for other in vivo applications. The reproducible viability obtained, despite the polydispersity in size, makes it a relevant tool for clinical translation in biotechnology and regenerative medicine. For the food industry, it may offer an effective way to protect microbes in adverse in vitro and in vivo environments, and it is promising for the high-throughput production of probiotics microencapsulation, as well as for the coadministration of probiotics and antibiotics.

## Figures and Tables

**Table 1:** Yield and encapsulation efficiency data.

Agitation Rate (rpm)	800	900	1000
Yield (%)	88 ± 3 ^a^	69 ± 3 ^b^	60 ± 3b ^b^
EE (%)	70 ± 6 ^c^	70 ± 13 ^c^	63 ± 14 ^c^

EE—encapsulation efficiency; values not assigned the same letters are significantly different (*p* < 0.05) by the Tukey–Kremer HSD test.

**Table 2:** Summary of the size distribution (µm).

Agitation Rate (rpm)	Mean ± SD (µm)	Median (µm)
800	222 ± 63.6	214
900	180 ± 51.8	169
1000	127 ± 30.7	123
